# Learning curve analysis of transvaginal natural orifice transluminal endoscopic surgery in treating ovarian cysts: a retrospective cohort study

**DOI:** 10.1186/s12905-024-03261-2

**Published:** 2024-07-25

**Authors:** Dan Feng, Tianjiao Liu, Xin Li, Lu Huang, Li Xiao, Li He, Yonghong Lin

**Affiliations:** 1 Department of Gynecology Chengdu Women’s and Children’s Central Hospital, School of Medicine, University of Electronic Science and Technology of China, No. 1617, Riyue Avenue, Chengdu, 610091 Sichuan People’s Republic of China; 2 Department of Obstetrics, School of Medicine, Chengdu Women’s and Children’s Central Hospital, University of Electronic Science and Technology of China, Chengdu, People’s Republic of China; 3 The Medical Administration Department, School of Medicine, Chengdu Women’s and Children’s Central Hospital, University of Electronic Science and Technology of China, No. 1617, Riyue Avenue, Chengdu, 610091 Sichuan People’s Republic of China; 4 No. 1617, Riyue Avenue, Chengdu, 611731 Sichuan People’s Republic of China

**Keywords:** Transvaginal natural orifice transluminal endoscopic surgery, Ovarian cystectomy, Learning curve, Cumulative sum analysis

## Abstract

**Background:**

Transvaginal Natural Orifice Transluminal Endoscopy (vNOTES) is regarded as a challenging surgical technique to learn but is promising in reducing perioperative pain and significantly improves the cosmetic outcomes. Previous studies on the learning curve analysis of vNOTES mainly focuses on the hysterectomy approach, while the vNOTES ovarian cystectomy’s learning curve was merely reported though more frequently performed than vNOTES hysterectomy. Therefore, this study seeks to analyze the learning curve of three surgeons with varying levels of experience in performing endoscopic surgery and vaginal surgeries for the treatment of ovarian cysts using vNOTES.

**Methods:**

A total of 127 patients with ovarian cysts of a variety of pathological types were treated by ovarian vNOTES performed by three surgeons of different levels of endoscopic and transvaginal surgical experience. Each surgeon’s learning curve was plotted using the Cumulative Sum method and divided into three or four phases of technique learning at the turning point of the learning curve. The sociodemographic and clinical features of patients in each phase were then compared and factors potentially associated with operation time were also screened.

**Results:**

The learning curve was presented in four phases. The operation time (OT) was significantly shorter in phases II (53.66 ± 16.55 min) and IV (54.39 ± 23.45 min) as compared with phases I (68.74 ± 15.85) and III (75.93 ± 30.55) (*p* < 0.001). More cases of serve pelvic adhesion and endometrioma were assigned in the later phases. The OT of endometriotic cysts had much longer than that of non-endometriotic cysts(62.57 ± 18.64 min vs. 49.88 ± 14.26 min, *p* = 0.15) The presence of pelvic adhesion [adjusted odds ratio (OR) 7.149 (0.506, 13.792), *p* = 0.035] and bilateral cyst [adjusted OR 16.996 (2.155, 31.837), *p* = 0.025], max diameter of cyst[adjusted OR 2.799 (0.174, 5.425), *p* = 0.037], and individual surgeon [adjusted OR -6.118 (-11.814, -0.423), *p* = 0.035] were significantly associated with OT.

**Conclusion:**

There learning curve of ovarian vNOTES has four phases. ovarian vNOTES could be mastered after performing seven, nine, and 16 cases by surgeons #1, 2 and 3 respectively, in gynecologic endoscopic surgeries.

**Trial registration:**

ChiCTR2200059282 (Registered on April 28th, 2022).

**Supplementary Information:**

The online version contains supplementary material available at 10.1186/s12905-024-03261-2.

## Background

Ovarian cysts are a prevalent condition that threatens the health of women of reproductive age as well as postmenopausal women. In the Chinese population, it reportedly has an occurrence rate of 4–7% [1]. It has been recommended by the American College of Obstetricians and Gynecologists that simple cysts found in ultrasonographic examinations should be treated conservatively and followed-up safely even in postmenopausal women. However, for symptomatic or non-simple cysts, timely surgical intervention is indicated to avoid rupture and other adverse outcomes such as torsion, hemorrhage and malignant degeneration.

The transvaginal natural orifice transluminal endoscopic surgery (vNOTES) is an emerging minimally invasive surgical (MIS) technique that reportedly has faster postoperative recovery, no visible abdominal skin scar, and easier specimen removal compared with traditional and even transumbilical laparoscopic single site surgery(TU-LESS) [2–4]. Since the first report of vNOTES ovarian cyst surgery in 2012, multiple clinical studies on its application in treating ovarian cysts have demonstrated its non-inferiority to laparoscopy in terms of surgical conversion and postoperative outcomes [2, 4–8]. Additionally, vNOTES has been approved feasible in repairing pelvic organ prolapse, treating ectopic pregnancy and achieving permanent female sterilization [9–13].

However, due to factors, including the totally different surgical approaches, opposite operating angles compared to traditional laparoscopy, narrow operating space, and chopstick effect (the instruments interfere with each other due to the narrow operating space in single port endoscopy), many specialists believe that the learning technique for vNOTES might be challenging, and the cost-effectiveness of learning the technique could be low [14, 15]. There is still debate regarding its cost-effectiveness and the suitable procedures for beginners [14–16]. Additionally, studies on the learning curve of such a complex new procedures often first report on surgeons with extensive experience in related techniques [17].

Based on our clinical practice in gynecology, we have found that ovarian vNOTES is relatively safe and we suppose that ovarian vNOTES is probably easier to learn compared to other procedures such as hysterectomy [18]. Thus, in this study, we aim to assess the number of cases required for three gynecologic surgeons with varying levels of experience in vaginal procedures and laparoendoscopic surgery to master ovarian vNOTES and evaluate the feasibility of learning this technique by plotting the learning curves and investigating their corresponding surgical outcomes.

## Methods

### Study design

The present study reviewed and analyzed the sociodemographic—age, body mass index (BMI), parity and gravity, previous delivery mode, etc.—and operation-related clinical features—surgical type, surgeons’ experience, pathological type, operation time (OT), estimated intraoperative blood loss, hemoglobin (Hb) decrease, cyst size, etc.—of 127 cases of ovarian vNOTES, which were performed between February 2019 and March 2023 by three gynecologic surgeons with different levels of experience of vaginal surgery at Chengdu Women’s and Children’s Central Hospital. We also analyzed the learning curves of each surgeon. Among the three surgeons, the most experienced one is an expert in laparoendoscopy and vaginal surgeries, who has 20 years of experience in gynecology and performed approximately 500 cases of TU-LESS and more than 50 cases of vaginal hysterectomy; the youngest surgeon in this study is a new attending physician who has 10 years of working experience and conducted more than 200 cases of TU-LESS but minimal case of vaginal hysterectomy; the other gynecologist has 15 years of working experience and successfully performed approximately 300 cases of TU-LESS and less than 50 cases of vaginal hysterectomy. Briefly, the most experienced surgeon was designated as surgeon #1; the surgeon with middle-level experience was designated as surgeon #2; and the least experienced surgeon was designated as surgeon #3. Among the clinical characteristics included in the present study, we mainly focused on the OT which reflected the competency of surgeons in performing ovarian vNOTES. We applied the cumulative sum (CUSUM) methodology on the OT to plot the learning curve of each surgeon and divided their vNOTES technique learning process into three or four phases at the turning points of the CUSUM curve, namely the exploration phase (Phase I), competence-acquiring phase (Phase II), challenge phase (Phase III), and proficiency phase (Phase IV, for the most highly experienced surgeon only). In the first and second phases, relatively easier surgeries were assigned. Since Phase III, more technically challenging surgeries based on findings of preoperative evaluations were assigned, especially to surgeon #1. The sociodemographic and clinicopathological characteristics of the cases performed in the different phases were also compared subsequently.

### Patient selection

The patients were included based on the following criteria:

1. has ovarian cysts which require surgical intervention;
2. has low possibility of malignancy according to imaging features and tumor markers;
3. shows a preference for vNOTES over other surgical options after being informed about the general procedures, postoperative wound recovery, complications, aesthetic characteristics, and economic costs of vNOTES, LESS, and traditional open surgery during the preoperative consultation.

The patients were excluded based on the following criteria:

1. has never had sexual intercourse;
2. has suspected or confirmed rectovaginal endometriosis;
3. has confirmed severe pelvic adhesion according to their medical history.
4. highly suspected severe pelvic adhesion according to physical examination and ultrasonic sliding sign;
5. active lower genital tract infection.
6. patients diagnosed with both endometriosis and infertility.

### Operating procedure of ovarian vNOTES

**Fig. 1:**
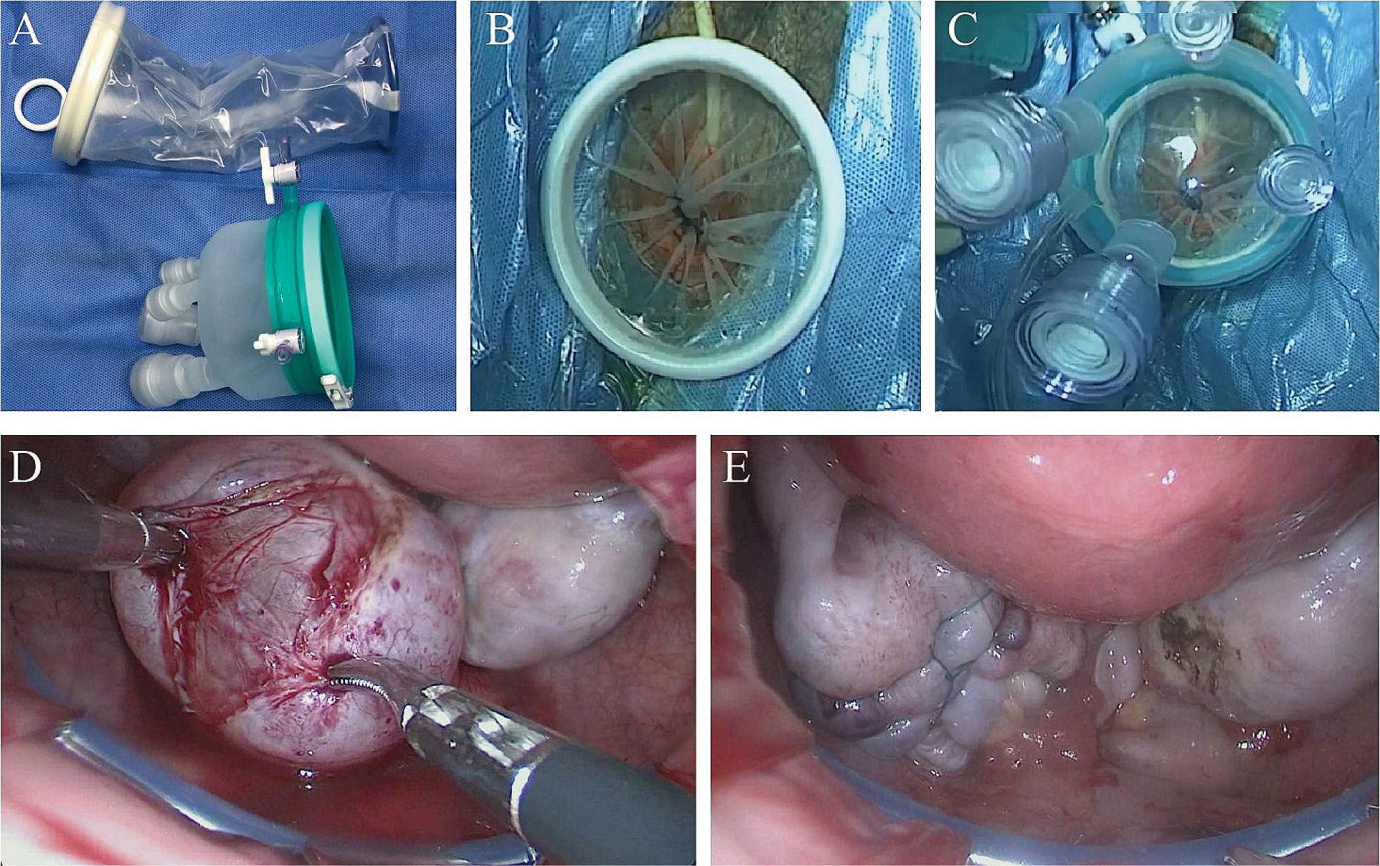
Key surgical steps of vNOTES ovarian cystectomy. (**A**) disposable retractor (**B**) insertion of the disposable retractor (**C**) establishment of the surgical platform (**D**) divesting the ovarian cyst wall (**E**) suture and reshaping of the ovary

This study applied similar surgical methods and equipment as described in previous publications [4, 19, 20]. The detailed surgical procedures were as follows:

1. Patients were placed in the Trendelenburg position and treated under general anesthesia following endotracheal intubation and insertion of Foley catheter for urinary drainage. After disinfection and draping, the cervix and vagina were exposed, especially the posterior fornix, which were disinfected three times.
2. The posterior labium of the cervix was pulled toward the upper and exterior direction using a cervical clamp to expose the posterior fornix of the vagina (Supplementary Fig. 1A). A 2–2.5-cm long posterior colpotomy incision was made at 0.5 cm below the cervical vaginal junction (Supplementary Fig. 1B) to get access to the abdominal cavity after incising the peritoneum (Supplementary Fig. 1 C and D).
3. A disposable multiple instrument access port (Beijing Aerospace Kadi Technology Development Institute, HK-TH-60.4TY) was placed into the abdominal cavity through the posterior culdotomy incision (Fig. 1A). The surgical platform was established after establishing the pneumoperitoneum (Fig. 1B C). The surgical visualization was achieved by applying a 10-mm, 30° endoscope (Karl Storz GmbH & Co. KG, Tuttlingen, Germany).
4. The abdominopelvic cavity was then carefully investigated to confirm the location, size, and adjacent organs of the ovarian cysts. Any pelvic adhesion, if existed, would be separated.
5. The cortex of the ovarian cyst was incised using scissor or monopolar electrotome. The cyst was then separated completely between the cyst wall and the rest of the ovarian stroma (Fig. 1D).
6. The excised cyst was incorporated into the bag, then the excised samples and bag were removed through the vaginal incision.
7. The ovary was sutured using a 2/0 or 3/0 absorbable suture line(VLOVL0315, Covidien Lic, Minneapolis, Minnesota, the United States) to stop bleeding and allow for reshaping (Fig. 1E).
8. Finally, the pelvic cavity was washed with sterile saline at body temperature, followed by the suction of CO_2_, removal of the retractor, and serial suturing of the vaginal incision using a 2/0 absorbable suture line (VLOVL0315, Covidien Lic, Minneapolis, Minnesota, the United States).

### Learning curve analysis

To analyze the changes in the surgeons’ proficiency in performing ovarian vNOTES during the study period, their two-dimensional learning curves were plotted using the following two parameters for each surgeon: X-value, which represents the number of ovarian vNOTES the corresponding surgeon has performed, which were ordered chronologically from the earliest to the latest, and Y-value, which indicates CUSUM_OT_. The CUSUM_OT_ of each case were calculated using the formula:

$$\:CUSUM\:OTn={\sum\:}_{i=1}^{n}\begin{array}{c}\\\:\left(\text{x}\text{i}-\mu\:\right)\\\:\end{array}$$

CUSUM_OT_ is the running total of differences between the individual case’s OT and mean OT of all cases. Hence, it could be conducted recursively. The OT of a certain case is designated as *xi*, and the mean OT of all cases is designated as *µ*. For instance, the CUSUM_OT1_ of case no.1 was the difference between the OT for the first case and µ. The CUSUM_OTn_ of case no. n is case no. (n – 1)’s CUSUM_OT(*n*−1)_ added to the difference between the OT for case no. n and µ. The calculation process was repeated until the final case was calculated, and CUSUM_OT final_ reaches zero [19, 21].

### Statistical analysis

IBM SPSS Statistics for Windows, version 25 (IBM Corp., Armonk, N.Y., USA) and Prism for Window, version 9.0 (GraphPad Software Inc., San Diego, CA, USA) were used for statistical analysis. Categorical variables are presented as numbers and percentages and were analyzed using Chi-squared test or Fisher Exact test when appropriate. As for continuous data, the normally distributed ones are shown as average ± standard deviation and compared using one-way analysis of variance. The non-normally distributed continuous variables are compared using the Kruskal–Wallis test. The learning curves of each surgeon were plotted using the aforementioned CUSUM methods. To screen out the parameters which were significantly associated with the OT, we established a multivariable linear regression model for OT and included all the relevant factors, such as age, BMI, previous pelvic surgery, pelvic adhesion, learning curve phase, estimated blood loss, uni- or bilaterality of cyst(s), occurrence complications, maximum cyst diameter, surgeon experience, parity, and pathologic types (endometrioma or not). A two-sided p-value lower than 0.05 was considered statistically significant.

## Results

### Overall profile of participants

Table 1 listed the general sociodemographic and perioperative information of all participants in our study. There were 127 patients with an average age of 35.52 ± 11.32 years and BMI of 21.83 ± 3.12 (kg/m2). The maximum diameter of their ovarian cysts was 5.33 ± 1.79 cm. Approximately 30% had undergone pelvic surgery before ovarian vNOTES. Approximately one-quarter (27 cases) experienced cesarean sections, and 51 cases (40.2%) had delivered vaginally. Approximately 30% of patients had pelvic adhesion, among which 22 cases (17.3%) were mild, 9 cases (7.1%) were average, and the remaining 12 cases (9.4%) were complicated with severe pelvic adhesion. Only 4 cases (3.1%) had ovarian endometriosis. Thirteen cases (10.2%) had bilateral cysts. The postoperative pathological diagnosis confirmed that there were 52 cases (40.9%) of teratoma, 39 cases (30.7%) of endometrioma, 20 cases (15.7%) of simple cyst, 16 cases (12.6%) of cystadenoma among all the cases. The patients had a postoperative hospitalization of 2.91 ± 0.93 days and intraoperatively lost 62.72 ± 93.93 mL blood.

**Table 1:** Patient characteristics and perioperative data (*N* = 127)

Variables*	Overall (*N* = 127)	Phase I (*N* = 31)	Phase II (*N* = 47)	Phase III (*N* = 31)	Phase IV (*N* = 18)	*P*-value
Age (years)	35.52 ± 11.32	33.58 ± 9.97	36.17 ± 11.31	34.55 ± 7.92	38.83 ± 17.20	0.422a
BMI (kg/m²)	21.83 ± 3.12	21.44 ± 2.39	21.62 ± 3.01	22.66 ± 3.65	21.65 ± 3.52	0.408a
Max diameter of cyst (cm)	5.33 ± 1.79	4.79 ± 1.39	5.11 ± 1.70	5.93 ± 1.96	5.78 ± 2.06	0.041a
History of pelvic surgery						0.738b
0	91 (71.7%)	24 (77.4%)	31 (66.0%)	24 (77.4%)	12 (66.7%)	
1	25 (19.7%)	5 (16.1%)	12 (25.5%)	5 (16.1%)	3 (16.7%)	
2	11 (8.7%)	2 (6.5%)	4 (8.5%)	2 (6.5%)	3 (16.7%)	
Previous delivery mode						0.582b
Cesarean section	27 (21.3%)	7 (22.6%)	11 (23.4%)	5 (16.1%)	4 (22.2%)	
Both	1 (0.8%)	0	0	1 (3.2%)	0	
Vaginal delivery	51 (40.2%)	13 (41.9%)	22 (46.8%)	9 (29.0%)	7 (38.9%)	
None	48 (37.8%)	11 (35.5%)	14 (29.8%)	16 (51.6%)	7 (38.9%)	
Presence of pelvic adhesion						0.591b
None	84 (66.1%)	25 (80.6%)	30 (63.8%)	18 (58.1%)	11 (61.1%)	
Mild	22 (17.3%)	5 (16.2%)	10 (21.3%)	6 (19.4%)	1 (3.2%)	
Middle	9 (7.1%)	1 (3.2%)	4 (8.5%)	3 (9.7%)	1 (3.2%)	
Severe	12 (9.4%)	0	3 (6.4%)	4 (12.9%)	5 (27.8%)	
Laterality of cyst(s)						0.457b
Unilateral	114 (89.8%)	26 (83.9%)	44 (93.6%)	27 (87.1%)	17 (94.4%)	
Bilateral	13 (10.2%)	5 (16.1%)	3 (6.4%)	4 (12.9%)	1 (5.6%)	
Postoperative pathological type						0.039b
Teratoma	52 (40.9%)	20 (64.5%)	17 (36.2%)	13 (41.9%)	2 (11.1%)	
Cystadenoma	16 (12.6%)	3 (9.7%)	6 (12.8%)	4 (12.9%)	3 (16.7%)	
Simple cyst	20 (15.7%)	4 (12.9%)	10 (21.3%)	3 (9.7%)	3 (16.7%)	
Endometrioma	39 (30.7%)	4(12.9%)	14(29.8%)	11(35.5%)	10(55.6%)	
Postoperative hospitalization (days)	2.91 ± 0.93	2.87 ± 0.67	2.81 ± 0.97	3.00 ± 0.52	3.11 ± 1.57	0.629a
Intraoperative blood loss (ml)	62.72 ± 93.93	44.51 ± 35.58	52.87 ± 53.92	90.97 ± 149.76	71.11 ± 115.40	0.202a
Postoperative pain score*						
Day 0	2.87 ± 0.34	2.90 ± 0.30	2.89 ± 0.31	2.80 ± 0.40	2.83 ± 0.38	0.624a
Day 1	2.10 ± 0.72	2.16 ± 0.64	2.11 ± 0.73	2.13 ± 0.85	1.94 ± 0.64	0.778a
Day 2	1.26 ± 0.63	1.35 ± 0.61	1.22 ± 0.66	1.23 ± 0.67	1.28 ± 0.57	0.805a
Day 3 **	0.70 ± 0.60	0.79 ± 0.56	0.76 ± 0.68	0.59 ± 0.50	0.61 ± 0.60	0.480a
Perioperative infection	3 (2.4%)	0	0	1 (3.2%)	2 (11.1%)	0.047b
Intraoperative complications***	3 (2.4%)	0	0	1 (3.2%)	2 (11.1%)	0.047b
Operation time (min)		68.74 ± 15.85	53.66 ± 16.55	75.93 ± 30.55	54.39 ± 23.45	< 0.001a

a Average and standard deviation

b One-way Analysis of Varianceb Number (percentage). Fisher Exact Test

*Visual Analogue Scale (VAS).

** Only 29, 37, 29, and 18 patients in Phase I, II, III, and IV respectively were included in the Day 3 VAS analysis due to the discharge of other patients

***1 blood transfusion, 1 paralytic ileus, 1 pelvic infection

### Learning curves of surgeons

**Fig. 2:**
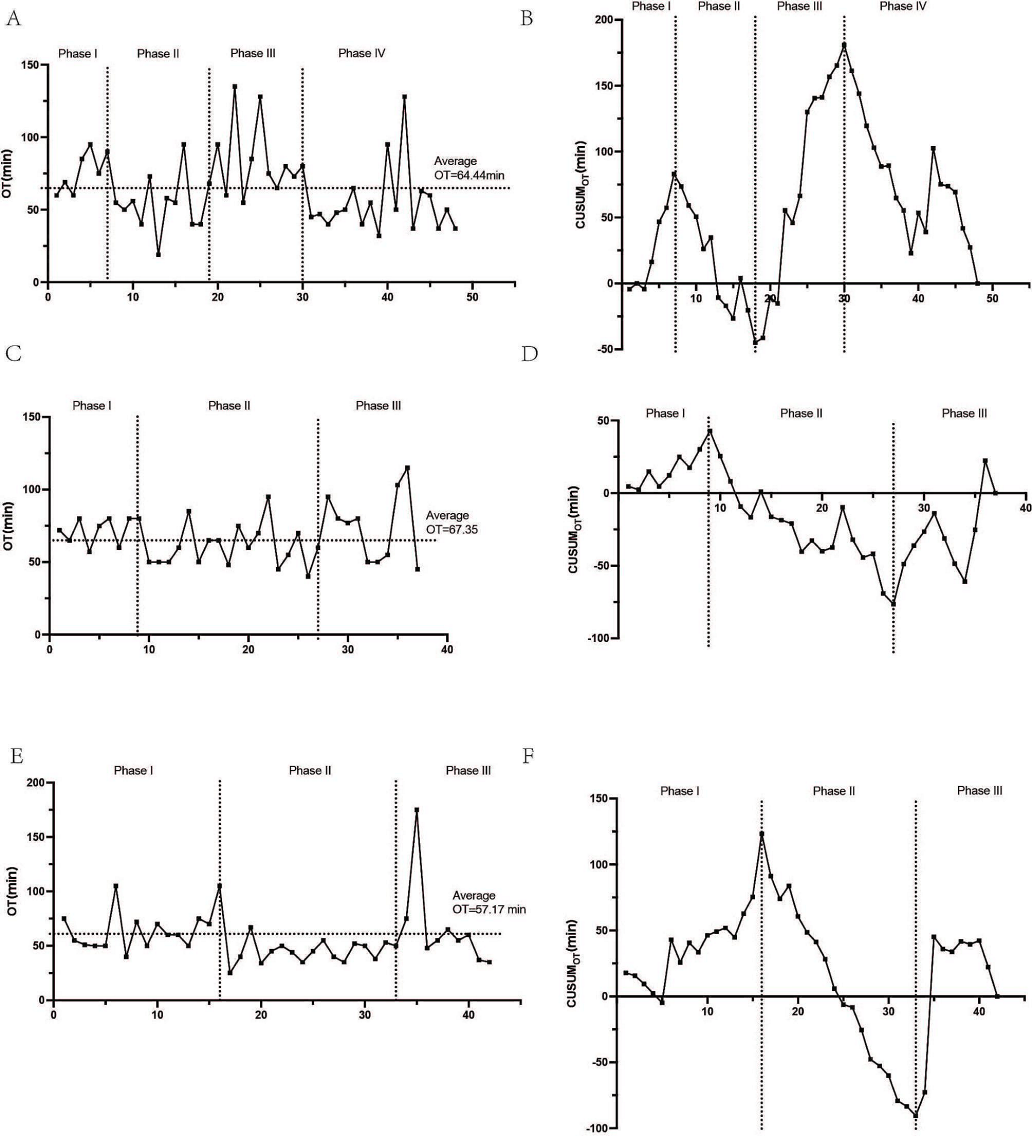
Learning curve analysis of vNOTES ovarian cystectomy performed by 3 surgeons. (**A**) The line graphs of OT-Case-number of surgeon #1; (**B**) The CUSUM-Case-number curve of surgeon #1; (**C**) The line graph of OT-Case-number of surgeon #2; (**D**) The CUSUM-Case-number curve of surgeon #2; (**E**) The line graph of OT-Case-number of surgeon #3; (**F**) The CUSUM-Case-number curve of surgeon #3

The line graphs of OT and CUSUM value along individual case’s number (in chronologic order) for each surgeon were plotted in Fig. 2A and B (surgeon #1), Fig. 2C and D (surgeon #2), Fig. 2E and F (surgeon #3). As shown in Fig. 2A, C and E, their average OT of surgeon #1–3 were 64.44 min, 67.35 and 57.17 min relatively. Generally, the surgeons spent longer than average OT in the exploration stage (Phase I) and shorter than average OT in the competency stage (Phase II). Consistently, we noticed that the CUMSUM curve of these three surgeons all presented an upward slope in Phase I and a downward slope in Phase II. A steep climbing trend in Phase I was observed in surgeon #1 (Fig. 2B), who performed 7 cases in the technique exploration stage (phase I). The surgeon #2 and #3 performed 9 and 16 cases respectively in the initial technique learning stage (Fig. 2D and F). As the surgeon and surgical team got more familiar with the ovarian vNOTES procedure, their operation time started to decrease since the beginning of Phase II and their CUSUM curve also showed a downward slope. Surgeon #1 performed 11 cases (case 8–18) in Phase II; while surgeon #2 and #3 both performed 18 cases (case 10–27) in Phase II. Since Phase III, more technically challenging cases were assigned to these surgeons, especially the most experienced one, making their learning curve regained the upward trend, though some fluctuation could also be observed in the Phase III of surgeon #2 and #3’s CUSUM-Case-number curve. As a result, surgeon #1 conducted 13 cases (case 18–30) in Phase III; while surgeon #2 conducted 11 cases (case 27–37); surgeon #3 performed 10 cases in the challenge phase (case 33–42). As more surgeries performed, a downward slope re-appeared in final stage of surgeon #1’s learning curve, “demarcating” a fourth phase (18 cases, case 31–48), during which surgeon #1 gradually gained surgical proficiency of ovarian vNOTES despite higher technical difficulty.

### Clinical characteristics of cases in the four stages

Then we compared the overall demographic and clinical features of participants in the four phases (Table 1). The comparisons of their age, BMI, gravidity, parity, delivery mode, previous history of abdominopelvic surgery, uni- or bilaterality of ovarian cysts, estimated intraoperative blood loss, Hb decrease at the 72nd postoperative hours, and postoperative pain score did not show any statistical significance. Meanwhile, we noticed a significantly shorter OT in phase II (53.66 ± 16.55 min) and IV (54.39 ± 23.45 min) as compared with phase I (68.74 ± 15.85) and III (75.93 ± 30.55) (*p* < 0.001). There were more cases with severe pelvic adhesion in later phases though not statistically significant [0 case in Phase I, 3 (6.4%) cases in Phase II, 4 cases (12.9%) in Phase III, and 5 cases (27.8%) in Phase IV, *p* = 0.591]. Similarly, there were significantly more cases with endometriosis (3 cases, 16.7%) in phase 4 than in phases I (1 case, 3.2%), II (0 case) and III (0 case) (*p* = 0.004). Moreover, the maximum cyst diameter significantly increased in later phases, with 4.79 ± 1.39 cm in phase I, 5.11 ± 1.70 cm and 5.93 ± 1.96 cm in Phase III, and 5.78 ± 2.06 cm in phase IV (*p* = 0.041). Similarly, the incidence of postoperative infection and other complications also increased remarkably in later phases, with 1 case (3.2%) of infection and blood transfusion occurring in phase III and two cases (11.1%) of infection, 1 case of paralytic ileus, and 1 pelvic infection in phase IV. Infection and other complications did not occur in phases I and II (*p* = 0.047).

Among all the 127 cases, there were 31 cases of vNOTES ovarian cystectomies performed in phase I, 47 cases performed in phase II, 37 cases in phase III, and 18 cases in phase IV(Table 1). We further divided the cases in each phase into four subgroups including the teratoma, cystadenoma, simple cyst, and endometrioma according to their pathologic types. The Fisher exact test showed that there were markedly less teratoma (2 cases, 11.1%) and more cholate cysts (10 cases, 55.6%) conducted in phase IV. Conversely, there was an opposite trend in ovarian vNOTES in treating more challenging pathologic types, the percentage of which climbed from 12.9% (4 cases) in phase I to 29.8% (14 cases) and 35.5% (11 cases) in phases II and III, respectively, to 55.6% (10 cases) in phase IV (*p* = 0.039).

Table 2 presents the OT of vNOTES in treating endometriotic cysts and non-endometriotic cysts in four distinct phases. A statistically significant difference in operation time between endometriotic cysts and non-endometriotic cysts is observed during Phase II, with mean times of 62.57 ± 18.64 min and 49.88 ± 14.26 min, respectively (*P* = 0.015). Additionally, across all phases, the operation time for non-endometriotic cysts shows a significant difference (*P* < 0.0001) as determined by one-way ANOVA.

**Table 2:** Operation time (in minutes) of endometrioticc cysts vs non- endometriotic cysts among the four phases stratified by pathologic disease

	Endometriotic cysts (*N* = 39)	Non-endometriotic cysts (*N* = 88)	*P*-value
Phase I (*N* = 31)	63.75 ± 12.50	69.48 ± 16.36	0.509a
Phase II (*N* = 47)	62.57 ± 18.64	49.88 ± 14.26	0.015a
Phase III (*N* = 31)	82.09 ± 29.20	72.55 ± 31.47	0.4146a
Phase IV (*N* = 18)	62.57 ± 30.04	49.18 ± 17.80	0.249a
P-value	0.206b	< 0.0001b	

a Student T test

b Average and standard deviation. One-way Analysis of Variance

### Multivariate regression analysis for operation time of ovarian vNOTES

**Fig. 3:**
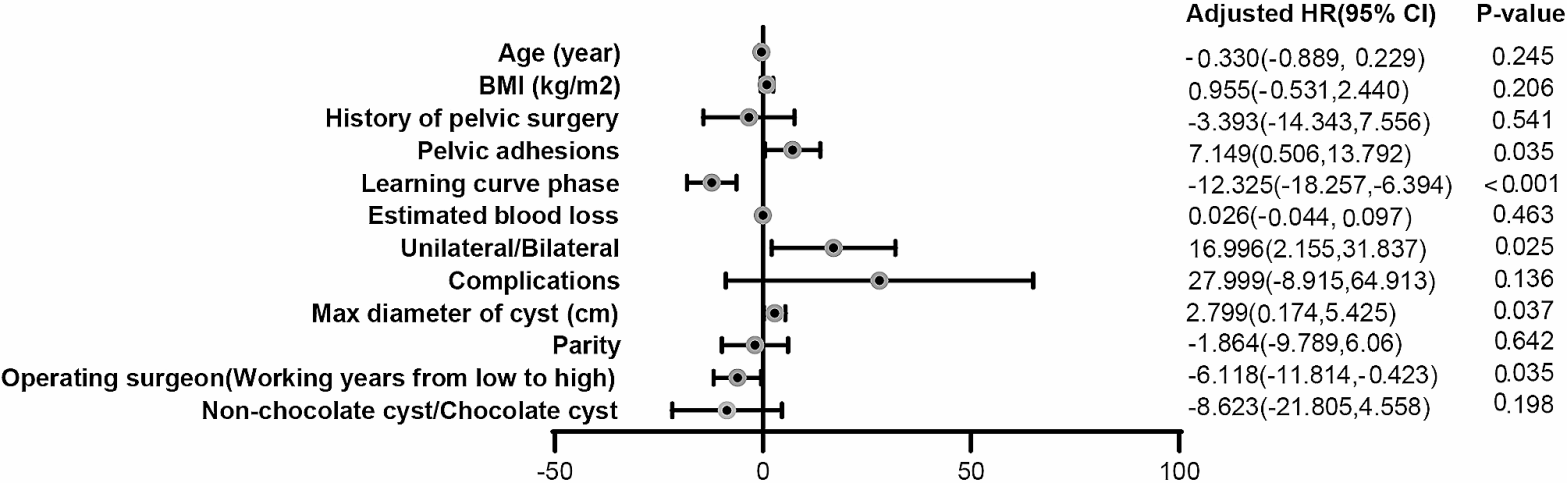
Multivariate regression analysis for operation time of vNOTES ovarian cystectomy

To further evaluate the potential significant association between OT and other factors, a multivariable linear regression model was established using the aforementioned methods. The results (Fig. 3) revealed that there are significant associations between OT and the following variables: (1) presence of pelvic adhesion [adjusted odds ratio (OR) 7.149 (0.506, 13.792), *p* = 0.035], (2) presence of bilateral cyst [adjusted OR 16.996 (2.155, 31.837), *p* = 0.025], (3) max diameter of cyst[adjusted OR 2.799 (0.174, 5.425), *p* = 0.037], and (4) surgeon who conducted the surgery [adjusted OR -6.118 (-11.814, -0.423), *p* = 0.035].

## Discussion

vNOTES is quite different from LESS and conventional laparoendoscopic surgery in multiple aspects. The establishment of a surgical path might be the most challenging one, due to the risks of injuring neighboring organs especially the rectum [22, 23]. More than 1,000 cases of ovarian vNOTES were conducted in our institute between December 2018 and October 2022 which were all performed through culdotomy in the posterior vaginal fornix [20]. After the initial technical exploration via approximately 30 cases of tentative vNOTES operation by two experts with more than 2 decades of experience in vaginal and laparoendoscopic surgeries, we established a standard operating procedure for conducting vNOTES and achieved better surgical outcomes and cosmetic satisfaction, faster recovery, and reduced postoperative pain compared with LESS or conventional laparoendoscopy [4, 19]. In this Standard Operating Procedure (SOP), we divested the cyst in the same manner as in LESS cystectomy after establishing the surgical platform. Our clinical practice of ovarian-vNOTES also supported the viewpoint that vNOTES is easier to perform compared to LESS because of the closer proximity to the ovaries via the culdotomy entrance, less severe chopstick effects, and easier specimen removal [22].

According to the international consensus of vNOTES experts, beginners should start from learning complete hysterectomy via vNOTES since the surgical path established therefrom would be much easier to access than the culdotomy in the posterior or anterior vaginal fornix [16]. Others state that vNOTES has a long learning process and may be quite challenging in inexperienced hands [23]. However, in our department, we observed that ovarian vNOTES is much more frequently conducted even by relatively less experienced hands due to the high incidence of ovarian and tubal diseases. Moreover, posterior vaginal fornix culdotomy is the dominantly preferred entrance for vNOTES surgery. Through a reasonably designed learning process (performing technically difficult cases in later stage or assigning them to experienced hands), our data showed that, even without experiencing vNOTES hysterectomy, surgeons with different levels of experience in performing laparoendoscopy could also master ovarian cystectomy via vNOTES in relatively less cases. The experienced surgeons only needed seven and nine cases, respectively, in the technique-acquiring phase, while the least experienced surgeons performed only 16 cases to reach competency, which supported the feasibility and promising prospect of promoting ovarian vNOTES. For young surgeons lacking experiences in conducting transvaginal surgeries, they could also complete ovarian cystectomy via vNOTES after standardized training. While it is also noteworthy that, given our city’s population of 20 million and our institution’s status as a tertiary maternal and child health care center, even our relatively less experienced gynecologic surgeons have performed a significant number of TU-LESS procedures.

Our study assigned relatively diverse pathologic types in the surgeons’ different learning phases, including the endometrioma. Whether endometrioma is suitable for vNOTES is still controversial. It is important to note that vNOTES is a novel and evolving procedure, with its indications and contraindications continuously updated as new clinical evidence emerges and equipment advances. The previous international expert consensus did not consider endometriomas and severe pelvic adhesions as contraindications for vNOTES [16]. One RCT of vNOTES for treating adnexal pathologies included patients with endometriomas [24], and another even included patients with severe pelvic adhesions [25]. It was also reported that many cases of endometriosis with severe adhesions can even be successfully managed using the vNOTES procedure without requiring conversion to another surgical approach [18]. Currently, due to the current technical limitations of vNOTES (limited surgical field which hinders the comprehensive assessment of the pelvic cavity), our institution mainly treats ovarian cysts with severe pelvic adhesions or infertility using TU-LESS or traditional laparoscopy. With the accumulation of surgical experience and advancements in instruments, such as the development of longer, softer, and more flexible endoscopes, we believe that the range of indications for vNOTES may gradually expand.

To date, there are many studies on the learning curve of vNOTES hysterectomy, while that of vNOTES ovarian cystectomy or adnexectomy was only reported once by Huang et al. [17, 19, 21, 26]. Compared with their study, our study had a similar cohort size but included more surgeons with different levels of gynecologic endoscopy experience. Moreover, we divided the learning curves into more stages, which could be a better reference for more surgeons. We also noticed that the mass diameter in their study was not significantly associated with the OT. In our study, cyst size and bilaterality are both positively associated with the OT, which might be caused by the different pathologic types included in our study. Another mismatch between our studies was that they did not find any cut-off point for determining the volume of cases needed to achieve mastery of adnexal vNOTES. Conversely, we noticed that seven to 16 cases were enough for initial technique establishment. This might also be explained by the various pathologic and surgeon types included in our study, given that all the cases were conducted by a single high-volume surgeon in their study. Nevertheless, our results both suggested that it might be more appropriate to start learning the vNOTES technique from ovarian or adnexal vNOTES, and less cases are needed to learn the technique and achieve proficiency than the vNOTES hysterectomy.

There are also several limitations in present study. Firstly, the surgeons who performed the vNOTES ovarian surgeries in our study may have performed more laparoscopic surgeries than many other less populous regions and countries, which may impair the generality of our findings. Secondly, the preoperative assessment of surgical difficulty was mainly made by subjective standards rather than standardized stratification or scoring system, which, to some extent, may hinder the assignment of surgeries to different surgeons and phases.

## Conclusions

Our learning curve analysis of ovarian vNOTES depicted four specific stages. vNOTES for ovarian cystectomy could be mastered after performing seven, nine, and 16 cases by surgeons with different endoscopic surgical experience. The presence of pelvic adhesion or bilateral cyst and cyst size were positively related to OT, while the surgeon’s experience was negatively correlated to OT.

### Electronic supplementary material

Below is the link to the electronic supplementary material.

Supplementary Material 1

## Data Availability

Data supporting the conclusion are provided within the manuscript or supplementary information files. The original data are available from the corresponding authors upon reasonable request.

## Abbreviations

vNOTES: Transvaginal natural orifice transluminal endoscopic surgery
MIS: Minimally invasive surgery
TU-LESS: Transumbilical laparoscopic single site surgery
OT: Operation time
BMI: Body mass index
CUSUM: Cumulative sum
Hb: Hemoglobin
