# Supplementary material for: Learning curve analysis of transvaginal natural orifice transluminal endoscopic surgery in treating ovarian cysts: a retrospective cohort study
Source: BMC Womens Health. 2024 Jul 25;24:424. doi: 10.1186/s12905-024-03261-2 (PMC11270853; doi:10.1186/s12905-024-03261-2)
Supplement: Supplementary file 1 — Supplementary Material 1 [file 12905_2024_3261_MOESM1_ESM.doc]

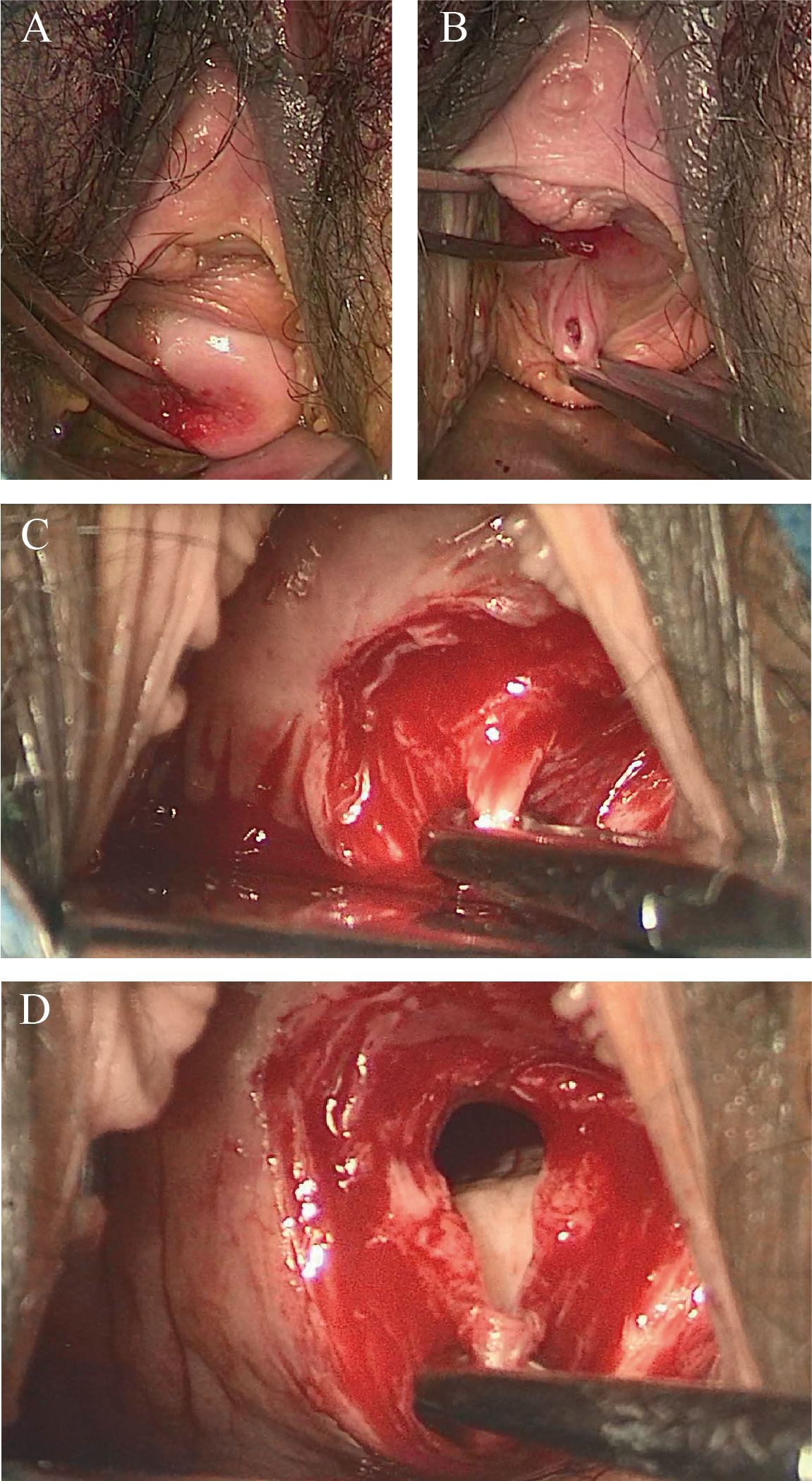


Supplementary Figure 1 Exposure of and the incision on the posterior fornix

(A) Exposure of the posterior fornix;

(B) Making of the posterior colpotomy incision;

(C) incising the peritoneum;

(D) opening of the peritoneum
